# Compressed sensing improved iterative reconstruction-reprojection algorithm for electron tomography

**DOI:** 10.1186/s12859-020-3529-3

**Published:** 2020-11-18

**Authors:** Lun Li, Renmin Han, Zhaotian Zhang, Tiande Guo, Zhiyong Liu, Fa Zhang

**Affiliations:** 1 High Performance Computer Research Center, Institute Of Computing Technology, Chinese Academy of Sciences, Beijing, 100190 China; 2 University of Chinese Academy of Sciences, Beijing, China; 3 Research Center for Mathematics and Interdisciplinary Sciences, Shandong University, Qingdao, 266237 People’s Republic of China; 4 National Natural Science Foundation of China, Beijing, 100085 China

**Keywords:** Electron tomography, Compressed sensing, Matching pursuit, Iterative reconstruction-reprojection

## Abstract

**Background:**

Electron tomography (ET) is an important technique for the study of complex biological structures and their functions. Electron tomography reconstructs the interior of a three-dimensional object from its projections at different orientations. However, due to the instrument limitation, the angular tilt range of the projections is limited within +70^∘^ to −70^∘^. The missing angle range is known as the missing wedge and will cause artifacts.

**Results:**

In this paper, we proposed a novel algorithm, compressed sensing improved iterative reconstruction-reprojection (CSIIRR), which follows the schedule of improved iterative reconstruction-reprojection but further considers the sparsity of the biological ultra-structural content in specimen. The proposed algorithm keeps both the merits of the improved iterative reconstruction-reprojection (IIRR) and compressed sensing, resulting in an estimation of the electron tomography with faster execution speed and better reconstruction result. A comprehensive experiment has been carried out, in which CSIIRR was challenged on both simulated and real-world datasets as well as compared with a number of classical methods. The experimental results prove the effectiveness and efficiency of CSIIRR, and further show its advantages over the other methods.

**Conclusions:**

The proposed algorithm has an obvious advance in the suppression of missing wedge effects and the restoration of missing information, which provides an option to the structural biologist for clear and accurate tomographic reconstruction.

## Background

Electron tomography (ET) is an important technique for the study of complex biological structures and their functions, which reconstructs the interior of a three-dimensional object from its projections at different orientations [1]. However, due to the instrument limitation, the angular tilt range of the projections is limited within +70^∘^ to −70^∘^. Such a series of the projections compose an angle-limited projection dataset, in which the unsampled high tilt angles are called the missing wedge and will cause artifacts. Therefore, the reconstruction of the tomogram in ET is an optimization problem with incomplete information. Additionally, the optimization problem becomes more difficult due to the extreme noise caused by the low electron doses in the collection of Electron Microscopy (EM) images.

Traditional 3D reconstruction methods, such as Weighted back-projection (WBP) and Filtered back-projection (FBP), suffer from the incomplete data and extremely noisy projections, resulting in the undesired artifacts. Many algorithms have been proposed to deal with the incompleteness caused by the limited projections and strong noise. By combining the algebra reconstruction technique (ART) and the nonlinear diffusion (ND) filter technique, filtered iterative reconstruction technique (FIRT) [2] could partially restore some information at the non-sampled angular region. Iterative reconstruction-reprojection (IRR) [3–5] and its variations, such as discrete IRR algorithm and the finite impulse response (FIR) lowpass filter (DIRRLF) [6], iterative reconstruction-reprojection (IRR) algorithm with total variation (TV) constraint (IRR-TV) [7], iterative reconstruction-reprojection in projection space (IRRP) algorithm [8] and algebraic iterative reconstruction-reprojection (AIRR) [9], estimate the missing projects by an iterative optimization procedure between the projection and image domain.

Improved iterative reconstruction-reprojection (IIRR) [10, 11] is one of the variations of the IRR algorithm, whose convergence under extremely noisy condition has been proved [12]. However, without prior information, IIRR is not efficient in suppressing the effect of the missing wedge and restoring the missing information for ET data, because of the low correlation between known projections and unknown projections in the missing wedge. On the other hand, other algorithms reduce artifacts by introducing prior information. Iterative Compressed-sensing Optimized Non-uniform fast Fourier transform reconstruction (ICON) [13–15] combines compressed sensing (CS) with non-uniform fast Fourier transform (NUFFT) in order to restore the missing information. There are two main steps in ICON. One is the fidelity preservation step which minimizes the difference between the reconstructed volume and the projections. Another one is prior sparsity restriction step which increases the sparsity of the reconstructed volume. Nonetheless, a drawback of ICON is its slow computational speed.

In this paper, we proposed a novel algorithm which follows the schedule of IIRR but further considers the sparsity of the biological ultra-structural content in specimen, resulting in an estimation of the electron tomography with faster execution speed and better reconstruction result. A comprehensive experiment on both the simulated and real-world dataset is carried out to estimate the performance of our algorithm and compare the new algorithm with other state-of-the-art methods. The experimental results indicate that the proposed algorithm has the ability to suppress the effect of missing wedge and restore the missing information within the missing wedge. Specifically, compared with the classical methods, our algorithm can achieve better reconstruction with faster convergence.

## Preliminaries

Without loss of generality, our following discussion is defined in two-dimensional space. Nevertheless, the algorithm we proposed can be extended to three-dimensional space easily.

### From filtered backprojection (FBP) to iterative reconstruction-reprojection (IRR)

The filtered back-projection (FBP) algorithm is one of the oldest algorithms in computing tomography. Its mathematical model could be concluded as following:

Given a function *f*(*x*) ($x\in \mathbb {R}^{2}$), the projections of function *f* is defined as a linear integral along a straight line $L=\{x\in \mathbb {R}^{2}|\left (\cos \alpha \right) x_{1}+\left (\sin \alpha \right) x_{2}=s\}$

1 $$ \begin{aligned} p(s,\alpha) & =\mathscr{R}f\\ & =\int_{L} \! f(x) \, \mathrm{d}x\\ & =\int_{0}^{\pi}\!f(t\sin\alpha+s\cos\alpha,-t\cos\alpha+s\sin\alpha)\, \mathrm{d}t, \end{aligned}  $$

where $\mathscr {R}$ is the Radon transform operator, $s\in \mathbb {R}$ is the distance of line *L* from the origin, *α* is the angle between normal vector to *L* and the *x*_1_ axis. Suppose the value of *p* on $\Omega \subset \{\left (s,\alpha \right)|\alpha \in \left (0,\pi \right ],s\in \mathbb {R}\}$ is known in prior. Let $\chi _{\Omega }(s,\alpha)=\left \{ \begin {array}{c} 1,(s,\alpha)\in \Omega \\ 0,(s,\alpha)\notin \Omega \end {array}\right.$ be the characteristic function of set *Ω*,*p*_*unknown*_(*s*,*α*)=(1−*χ*_*Ω*_(*s*,*α*))*p*(*s*,*α*) be the unknown projections and *p*_*known*_(*s*,*α*)=*χ*_*Ω*_(*s*,*α*)*p*(*s*,*α*) be the known projections. FBP will first apply a filter on the projections and then backproject these filtered projections to get an estimation of the original signal *f*:

1) Convolution (Filtering)

2 $$ \begin{aligned} p_{c}(s,\alpha)&=p_{known}(s,\alpha)*\mathscr{F}_{1}^{-1}|\omega| \\ &=\int p_{known}(t,\alpha)\cdot (\mathscr{F}_{1}^{-1}|\omega|)(s-t)dt \end{aligned}  $$

where * denotes convolution, $\mathscr {F}_{1}^{-1}$ denotes the one dimensional Fourier transform.

2) Backprojection

3 $$ \begin{aligned} \widetilde{f}(x_{1},x_{2})&= \mathscr{B}p_{c}\\ &=\int p_{c}(x_{1}\cos\alpha+x_{2}\sin\alpha,\alpha)d\alpha, \end{aligned}  $$

where $\widetilde {f}$ is the estimation of *f*, *x*_*i*_ is the *i*th element of *x*, $\mathscr {B}$ is the backprojection operator. It should be noted that, in FBP, the unknown projections *p*_*unknown*_ have been set to 0.

Meanwhile, the iterative reconstruction-reprojection algorithm (IRR) is an iterative reconstruction method which is able to suppress the effect of insufficient tilt angles by estimating the unknown projections. At the beginning, all the unknown projections in the system $p_{unknown}^{1}$ are set to 0 as in FBP. During the computational process of IRR, for the *i*th iteration, the *i*th estimation of *f* will be reconstructed from both the known projections *p*_*known*_ and the *i*th estimated unknown projections $p_{unknown}^{i}$:

4 $$ f^{i}(x_{1},x_{2})=\mathscr{B}((p_{known}+p_{unknown}^{i})*\mathscr{F}_{1}^{-1}|\omega|).  $$

However, indeed, we do not know the unknown projections $p_{unknown}^{i}$. In IRR, the (*i*)th unknown projections are estimated from *f* in the previous iteration:

5 $$ p_{unknown}^{i}(s,\alpha)=(1-\chi_{\Omega})\mathscr{R}f^{i-1}.  $$

By estimating the unknown projections rather than setting them to 0, IRR suffers less from the ’missing wedge’ problem than FBP.

### The improved algorithms based on IRR

#### Improved iterative reconstruction-reprojection (IIRR)

A drawback of IRR is that the convergence of IRR under extremely noisy condition is not guaranteed. By introducing a relaxed parameter *λ* into IRR, the convergence under extremely noisy condition has been proved. More precisely, IIRR changes Eq. (4) into

6 $$ f^{i}(x_{1},x_{2})=\mathscr{B}((p_{known}+\lambda p_{unknown}^{i})*\mathscr{F}_{1}^{-1}|\omega|),  $$

where 0<*λ*<1 is a relaxed parameter. The smaller value of *λ* considers more in the known projections while larger value of *λ* considers more about the unknown projections. IIRR can restrain the artifact caused by the incomplete data in Computerized Tomography (CT).

#### Iterative reconstruction-reprojection with total variation (IRR-TV)

IRR-TV is an reconstruction method which is developed for few-view projections, in which the angular tilt range of the projections is not limited but the number of projections is limited. IRR-TV poses the reconstruction problem as an optimization problem with the form as:

7 $$ \begin{array}{c l} \min\limits_{f} & TV(f)\\ s.t. & Af=b, \end{array}  $$

where *Af*=*b* is the fidelity term,

8 $$ \begin{aligned} {TV(f)=}{\sum_{(x_{1},x_{2},x_{3})\in\mathbb{Z}^{3}}((f_{x_{1},x_{2},x_{3}}-f_{x_{1}-1,x_{2},x_{3}})^{2}} {+(f_{x_{1},x_{2},x_{3}}-f_{x_{1},x_{2}-1,x_{3}})^{2}+\left(f_{x_{1},x_{2},x_{3}}-f_{x_{1},x_{2},x_{3}-1})^{2}\right)^{\frac{1}{2}}} \end{aligned}  $$

is the total variation norm, *f* is the unknown 3D reconstruction volume, $\left (x_{1},x_{2},x_{3}\right)\in \mathbb {Z}^{3}$ is the coordinate of voxels in unknown volume, *A* is the projection matrix, and *b* is the observations or projections. By introducing TV into IRR, IRR-TV achieves an accelerated convergence rate compared with the classic IRR algorithm.

Specifically, in each iteration before the estimation of unknown projections, IRR-TV minimizes the TV norm by deepest gradient descent:

9 $$ f^{i}=f^{i}-d\cdot\frac{\partial TV(f^{i})}{\partial f^{i}},  $$

where *d* is step size, *f*^*i*^ is the *i*th estimation of *f*, and *TV*(·) is the TV norm.

### Matching pursuit

Matching pursuit (MP) is an algorithm which represents a signal in a redundant basis. Let $\mathscr {D}=\{g_{\gamma }\}_{\gamma \in \Gamma }$ be a family of vectors in Hilbert space and *f* be a signal. *f* can be represented as a linear combination of *N* vectors selected from $\mathscr {D}$

10 $$ f\approx\sum_{n=1}^{N}a_{\gamma_{n}}g_{\gamma_{n}},  $$

where $a_{\gamma _{n}}$ is the weight of $g_{\gamma _{n}}, g_{\gamma _{n}}$ is the vectors selected from $\mathscr {D}$, *N* is the total number of vectors selected from $\mathscr {D}$. The basic idea of MP is choosing vectors from $\mathscr {D}$ greedily. More precisely, let *R*_1_=*f* be the initial approximation error. For *i*=1,2,3,..., MP finds $g_{\gamma _{i}}$ with maximum absolute value of inner product $\phantom {\dot {i}\!}|<R_{i},g_{\gamma _{i}}>|$, where $\phantom {\dot {i}\!}R_{i}=f-\sum _{n=1}^{i}a_{\gamma _{n}}g_{\gamma _{n}}$ is the *i*th approximation error. Then we set $\phantom {\dot {i}\!}a_{i}=<R_{i},g_{\gamma _{i}}>$. The process continues until the approximation error is small enough.

## Methods

### Problem definition

Despite the success of IIRR in Computed Tomography (CT), IIRR is not efficient in suppressing the effect of the missing wedge and restore the missing information for ET data. Here, an algorithm based on the schedule of IIRR with the consideration of sparse information is proposed to achieve better ET reconstruction.

According to the previous study [13], the biological ultra-structural content is relatively sparse with respect to the surrounding solvent. Such sparsity is used as the prior information in our proposed method. Formally, let *f*(*x*) be the space function ($x\in \mathbb {R}^{2}$) representing the sample and suppose that *f* is supported on set *T* which is a subset of $\mathbb {Z}^{2}$. The consequent optimization problem could be defined as

11 $$ \begin{array}{c l} \min\limits_{f} & \Vert f\Vert_{0} \\ s.t. & \chi_{\Omega}\mathscr{R}f=p_{known}, \end{array}  $$

where $\Vert f\Vert _{0}=\#\{x\in \mathbb {Z}^{2}\cap T|f(x)\neq 0\}$ is the *l*_0_ norm of *f*, x is a 2 dimensional vector representing the coordinate of pixels in picture *f*, $\mathscr {R}$ is the Radon transform, *p*_*known*_ is the known projections, *χ*_*Ω*_ is the characteristic function of set *Ω*, and *Ω* is the subset of projection space corresponding to the known projections.

### Compressed sensing improved iterative reconstruction-reprojection (CSIIRR)

We designed a new algorithm to solve the optimization problem (11) by combining IIRR and CS. The proposed algorithm is elaborated as follows.

- Step 0: Preprocess
- Step 1: Estimate the unknown projections
- Step 2: Reconstruct via modified matching pursuit (MMP), return to step 1

#### Step 0: preprocess

Alignment is needed for the raw projections before reconstruction for ET data [16, 17]. Then we need an initial guess of specimen *f* which can be the reconstruction result via any reconstruction method or even a zero function. We denote the initial guess of *f* as *f*^0^.

#### Step 1: estimate the unknown projections

Let *f*^*k*−1^ be the reconstruction result in the (*k*−1)th step, *k*=1,2,.... We estimate the unknown projections by reprojecting *f*^*k*−1^ at the corresponding tilt angles. We denote the estimated projections as

12 $$ p_{unknown}^{k-1}=(1-\chi_{\Omega})\mathscr{R}f^{k-1},  $$

where the notation $\mathscr {R}$ denotes the Radon transform, *Ω* is the subset of projection space corresponding to the known projections, and *χ*_*Ω*_ denotes the characteristic function of *Ω*.

#### Step 2: reconstruct via modified MP (MMP)

In this step, we want to reconstruct specimen *f*^*k*^ from the known projections *p*_*known*_ and the estimated projections $p_{unknown}^{k-1}$. We seek for the sparsest *f*^*k*^ such that $\mathscr {R}f^{k-1}=p_{known}+\lambda p_{unknown}^{k-1}$, where 0<*λ*<1 is a relaxed parameter. A small *λ* is suggested for extremely noisy data because the reliability of estimated unknown projections is low with high noise levels. So we introduced compressed sensing to this step by a modified matching pursuit method. The detailed process of our modified matching pursuit method is described in Algorithm 1.


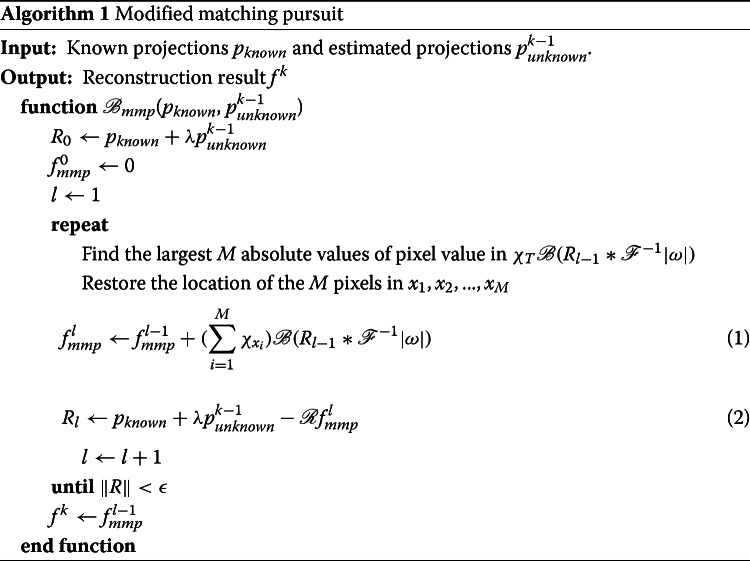


As shown in Algorithm 1, we first initialize the approximation of reconstruction result $f_{mmp}^{0}$ to zero vector, and the residual *R*_0_ to $p_{known}+\lambda p_{unknown}^{k-1}$. In the *l*-th iteration, we want to find the largest M absolute values of pixel value in $\chi _{T}\mathscr {B}(R_{l-1}*\mathscr {F}^{-1}|\omega |)$ and restore the location of the *M* voxels in *x*_1_,*x*_2_,...,*x*_*M*_. The parameter *M* affects the sparsity of the reconstruction result. A small *M* is suggested if the biological ultra-structural content is sparse in the specimen. Next, we use the restored information to update the approximation $f_{mmp}^{l}$ by Eq. (??) and the consequent residual *R*_*l*_ by Eq. (??). The process continues until ∥*R*∥<*ε*. After obtaining *f*^*k*^, the algorithm returns to step 1 in order to optimize the estimated projections $p_{unknown}^{k}$.

The main advantage of combining CS with IIRR is to introduce sparsity into IIRR. There are two main differences between the Algorithm 1 and MP methods. First, Algorithm 1 estimates the tomogram not only from the known projections *p*_*known*_ but also from the estimated projections $p_{unknown}^{k-1}$, which makes the proposed algorithm more stable than MP. Second, instead of choosing only one atom, Algorithm 1 chooses multiple pixels in each iteration. By doing so, Algorithm 1 can achieve a quick convergence compared with the classic MP. Moreover, there are also some differences between IRR-TV and CSIIRR. First, the two methods choose different regularization. IRR-TV minimizes the TV norm while CSIIRR minimizes the *l*_0_ norm. Second, IRR-TV adds a ’deepest gradient descent’ step after the back projection, which is time-consuming. Third, IRR-TV focuses on the few-view problem in CT while CSIIRR focuses on the angle-limited problem in ET. Especially, CSIIRR is able to handle extremely noisy data on which IRR-TV usually fails.

## Results

The CSIIRR is challenged by both the simulated data and real-world data. Expect for CSIIRR, the filtered back-projection (FBP), IIRR, weighted back-projection (WBP), Iterative Compressed-sensing Optimized Non-uniform fast Fourier transform reconstruction (ICON) [13, 14] and total variation (TV) are also tested in our experiment as a comparison. (Here, the algorithms of FBP, IIRR and CSIIRR are implemented with MATLAB, the implementation of ICON is download from http://ear.ict.ac.cn and the implementation of WBP comes from the ’tilt’ module in IMOD.) In the experiment, the relaxation factor *λ* is set to 0.99 for both IIRR and the CSIIRR.

### Performance on simulated data

#### Overall evaluation

First, CSIIRR is challenged on the noise-free simulated data. The ribosome structure downloaded from EMDB-3489 [18] is used as the ground-truth, whose volume size of EMDB-3489 is 400^3^ voxels. A set of the projections with the range of the tilt angle from 60^∘^ to −60^∘^ is generated as the measured observations, with an angular increment of 1^∘^.

To reconstruct the tomogram, the number of iterations is set to 10 in IIRR. For the sake of fairness, the number of outer-loop iterations in our proposed algorithm is also set to 10. To make reconstruction results converge, the number of iterations for modified MP in the proposed algorithm is set to 50. To trade off the convergence rate of CSIIRR and the sparsity of the output, the number of atoms selected in each iteration (*M* in Algorithm 1) is set to 335103 in modified MP.

**Fig. 1:**
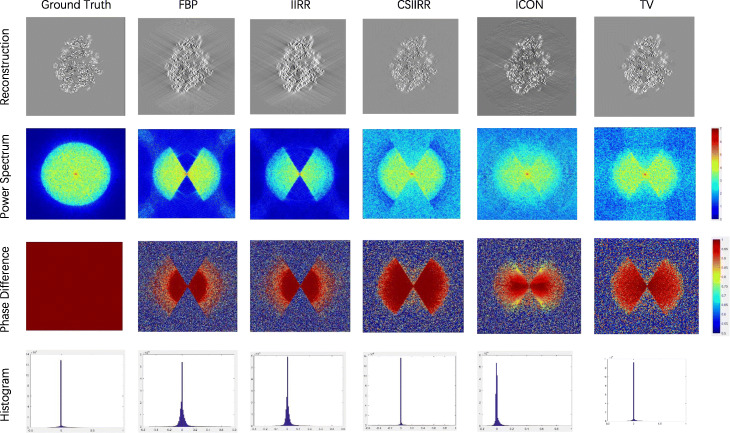
Missing wedge analysis. Comparison of central slices from the ground truth and the tomograms reconstructed by FBP, IIRR, CSIIRR, ICON, TV, of the log-scaled power spectrum, phase difference, and histogram

Figure 1 demonstrates the reconstruction results obtained by FBP, IIRR, CSIIRR, ICON and TV. As shown in the first row, the central slice of the reconstruction produced by CSIIRR suffers the least artifacts caused by the incompleteness of the sampling angle compared with the ones of other methods. The log-scaled power spectrums on the second row of Fig. 1 shows that CSIIRR has filled some missing data in the missing wedge. Furthermore, the phase difference of the tomograms reconstructed by different methods has been presented in the third row of Fig. 1 (The point-to-point phase difference [13] *Δ* of *a* and *b* is defined as $\Delta =\vert \frac {\vert a-b\vert }{\pi }-1\vert $.), which shows that CSIIRR has restored some correct information in the missing wedge. Moreover, the histograms demonstrated in the fourth row of Fig. 1 further support the conclusion made from the log-scaled power spectrum, in which the histogram shows that the reconstruction result by CSIIRR has a much more similar voxel distribution with the ground-truth, compared with the ones obtained from other methods. Additionally, the histogram accurately presents the sparseness of the reconstruction generated by CSIIRR.

#### Convergence analysis

**Fig. 2:**
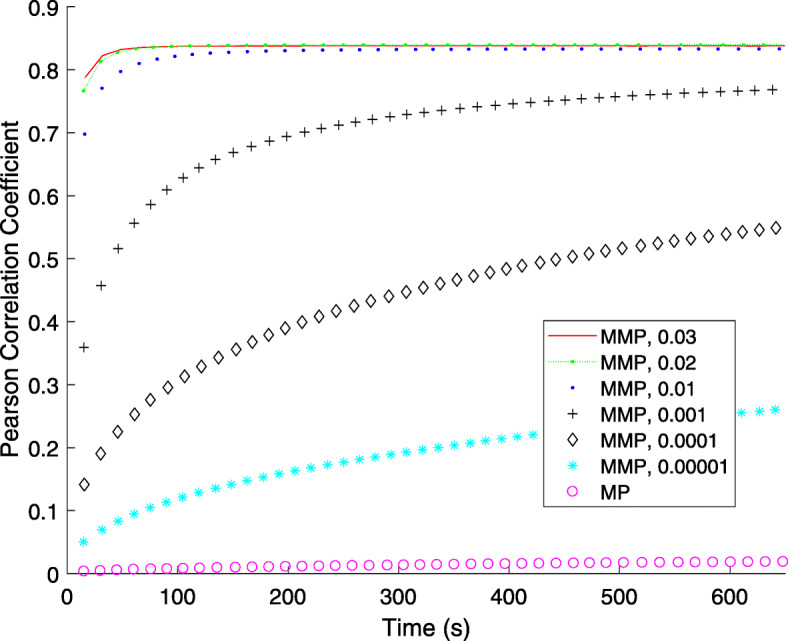
Speed acceleration gained by modified MP. The PCC of MP and modified MP (MMP) with respect to running time. The number in the legend represent the ratio of *M* to the number of volumes in *f*. *M* is the number of pixel values updated in each iteration

Except for the reconstruction accuracy, the convergence and running speed are also the important issues for a novel iterative method. Here, we first investigated the convergence of the modified MP algorithm (MMP) and the classic matching pursuit algorithm (MP) under different parameter configuration. And then compared the runtime of the proposed CSIIRR with the other classical methods (e.g. WBP, IIRR and ICON). The simulated data shown in the previous section is used as the benchmark dataset. When comparing the proposed modified MP and classic MP, we omitted the estimation of unknown projection in MMP (i.e., we set $p_{unknown}^{k-1}=0$ in Algorithm 1) for fair comparison. One point should be noted is that the classic MP is a special case of MMP if setting the parameter *M* to 1. The Pearson correlation coefficient (PCC) between the ground-truth and each turn’s reconstruction result is measured to indicate the improvement of reconstruction quality during the iteration process. Figure 2 demonstrates the change of PCC value for the two methods. It can be found that the MMP algorithm has a much faster convergence rate compared with the classic MP algorithm, no matter how we set the parameters. Furthermore, in the early iterations, the larger the *M* is, the faster the convergence speed will be.

**Fig. 3:**
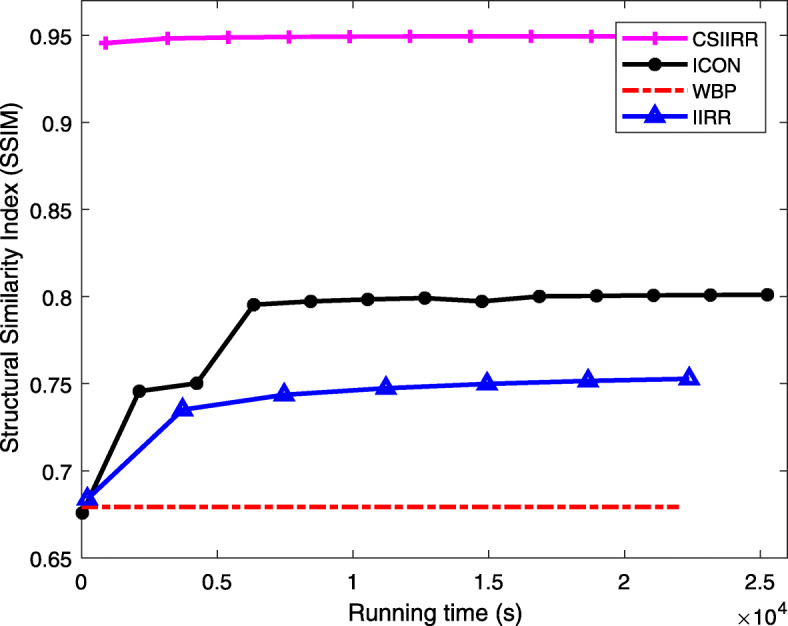
Comparing of running time. The SSIM of CSIIRR, ICON, WBP and IIRR with respect to the running time

When comparing the run-time between the whole CSIIRR framework and the classic WBP, IIRR, and ICON methods, the *M* used in modified MP is set to 335103 (i.e., with 0.01 ratio). We use structural similarity (SSIM) which is a measurement of image quality to evaluate CSIIRR. Figure 3 shows the experimental result, in which the SSIM curves of CSIIRR, ICON, WBP and IIRR with respect to the running time are demonstrated. It can be observed that CSIIRR converged to the highest SSIM value with the fastest convergence rate.

#### Effect of tilt angle change

Next, we tested the robustness of CSIIRR on different tilt angle ranges and intervals.

**Fig. 4:**
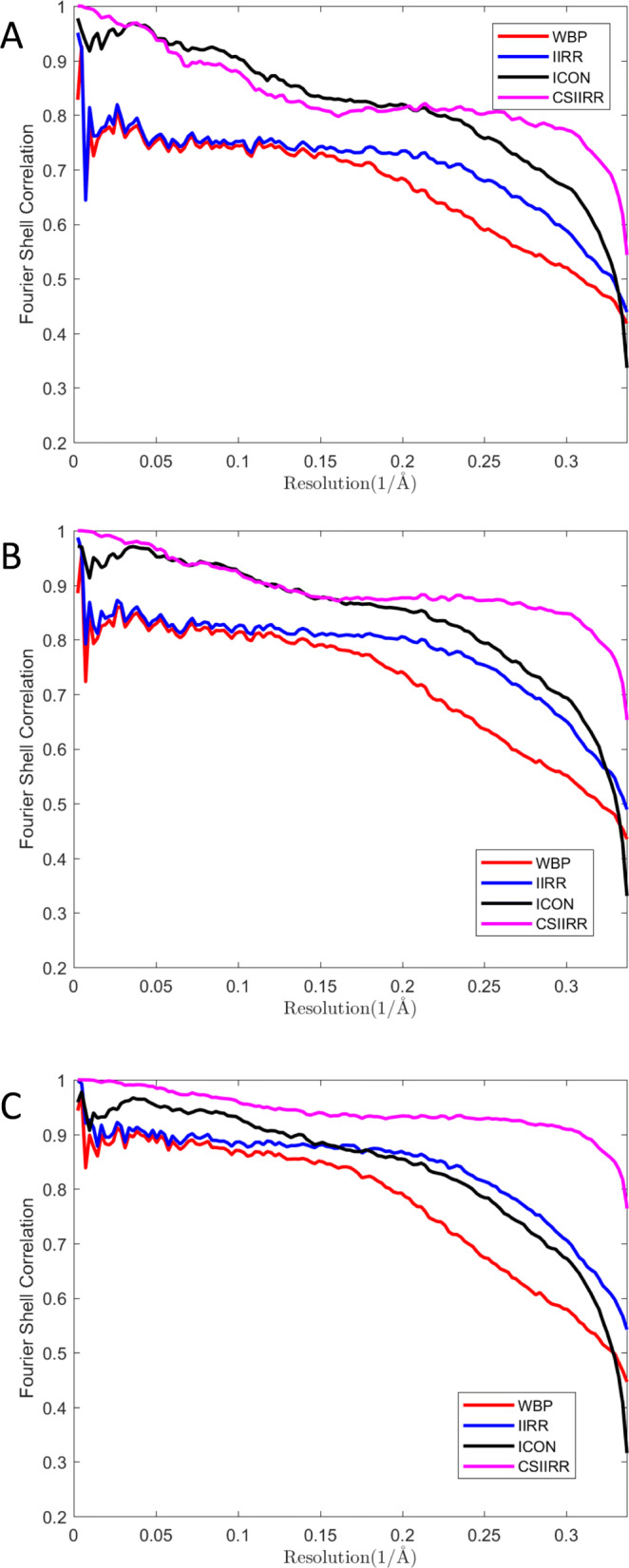
Effect of angle range. **a**, **b**, **c** is the FSC curves of tomograms reconstructed from simulated data with angular range set to −50^∘^ to 50^∘^,−60^∘^ to 60^∘^ and −70^∘^ to 70^∘^ respectively

CSIIRR was tested on simulated data with angular range set to −50^∘^ to 50^∘^,−60^∘^ to 60^∘^ and −70^∘^ to 70^∘^ (Fig. 4). As shown in Fig. 4, CSIIRR’s FSC curve is significantly higher than the ones of other methods.

**Fig. 5:**
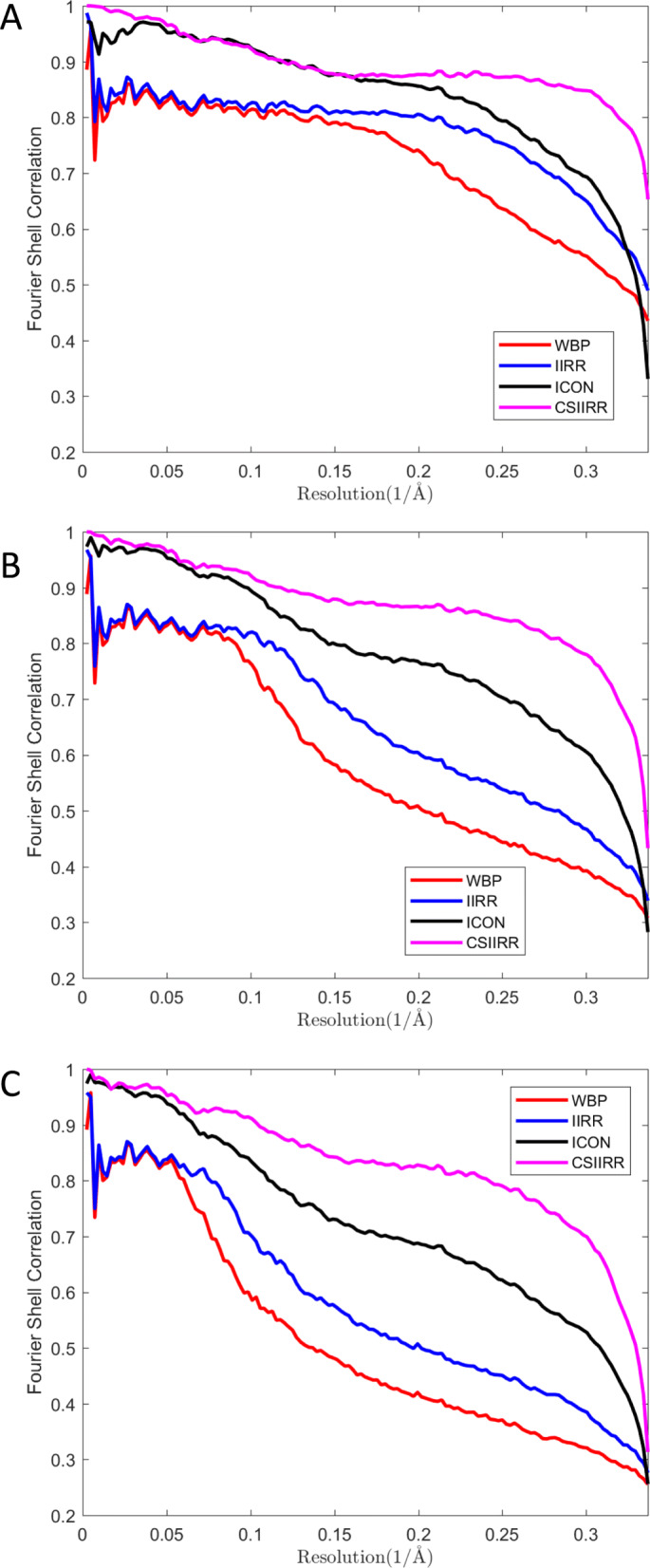
Effect of angular increments. **a**, **b**, **c** is the FSC curves of tomograms reconstructed from simulated data with angular increments set to 1^∘^,2^∘^ and 3^∘^ respectively

Furthermore, CSIIRR was tested on simulated data with angular intervals 1^∘^,2^∘^ and 3^∘^ (Fig. 5). As shown in Fig. 5, CSIIRR’s FSC curve has significantly higher coefficients than that of other methods, especially for high frequency.

#### Effect of noise

**Fig. 6:**
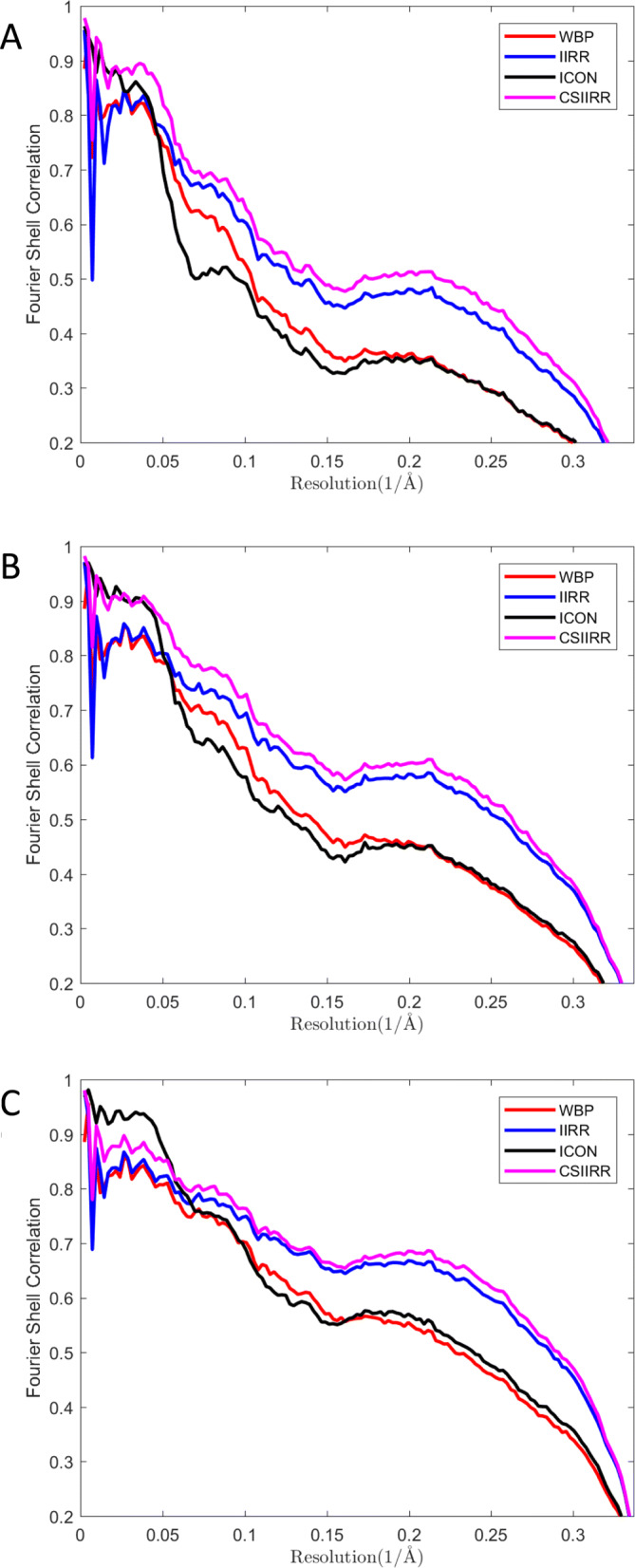
Effect of noise. **a**, **b**, **c** is the FSC curves of tomograms reconstructed from simulated data with SNR set to 0.5, 1 and 2 respectively

The effect of noise on the behavior CSIIRR has been investigated. The noise with signal-to-noise ratio *SNR*=0.5,1,2 is added to the projections and the tomogram is reconstructed by WBP, IIRR, ICON and CSIIRR. The Fourier shell correlation (FSC) between the reconstructed tomogram and the ground-truth is calculated to show the reconstruction quality of each method. Figure 6 shows corresponding FSC curve. For the data with *SNR*=0.5 and 1(Fig. 6a, b), the result obtained by CSIIRR achieves the highest correlation value for each Fourier frequency compared with the ones of WBP, IIRR and ICON. For the data with *SNR*=2 (Fig. 6c), the result obtained by CSIIRR achieves the highest correlation value for high frequency compared with the ones of WBP, IIRR and ICON.

### Performance on real data

The proposed CSIIRR was further challenged by real-world datasets. We first made a whole tilt series reconstruction to see the behavior differences between the proposed CSIIRR and the other classical methods (e.g., IIRR and ICON). Then, a leave-one-out analysis was carried out to further make a quantitative analysis.

The leave-one-out [19] validation can show how precise the missing information is restored by a reconstruction method. In detail, we exclude the projection with the minimum tilt angle from the tilt series and then reconstruct the tomogram without considering the excluded projection. After the reconstruction, we reproject the tomogram according to the tilt angle value of the excluded projection and calculate the similarity between the reprojection and the exact excluded projection by Fourier ring correlation.

#### Experiment on centriole

The Centriole dataset is a tilt series of plastic embedded cell section around a centriole region, which was taken on a FEI TF30 microscope (operated at 300 kV) with a Gatan Camera. This dataset is downloaded from IMOD’s website [20]. The tilt angles of the projections range from +65.0^∘^ to −61.0^∘^ at 2^∘^ intervals. The size of each projection is 1024×1024 with a pixel size of 1.01 nm. The tilt series is aligned in advance by IMOD [20].

To reconstruct the tomogram, the number of iterations is set to 15 in IIRR. For the sake of fairness, the number of outer-loop iterations in our proposed algorithm is also set to 15. The number of iterations for modified MP in CSIIRR is set to 3, and the number of atoms selected in each iterations M is set to 19,191,398 in modified MP. The iteration is set to ’30,120,300’ in ICON.

**Fig. 7:**
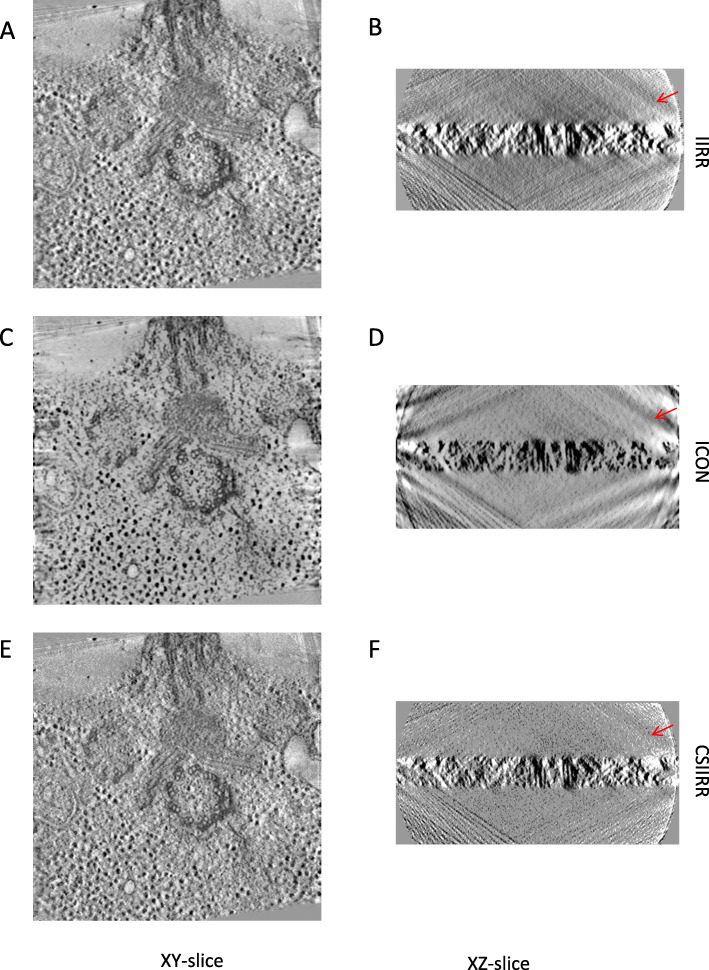
Reconstruction result of the Centriole dataset. **a**, **c**, **e** is the XY-slice of reconstruction result from IIRR, ICON and CSIIRR respectively. **b**, **d**, **f** is the XZ-slice of reconstruction result from IIRR, ICON and CSIIRR respectively

Figure 7 shows the XY-slice and XZ-slice of the reconstructed tomograms by different methods. From Figs. 7a, b and e, f, it can be found that the tomogram reconstructed by CSIIRR has a clearer background comparing with the ones reconstructed by IIRR. Additionally, according to Fig. 7c, d, stripe noise appears around the boundary of the XY-slice of tomogram from ICON while this kind of stripe noise does not appear around the boundary of the XY-slice of tomogram from CSIIRR.

**Fig. 8:**
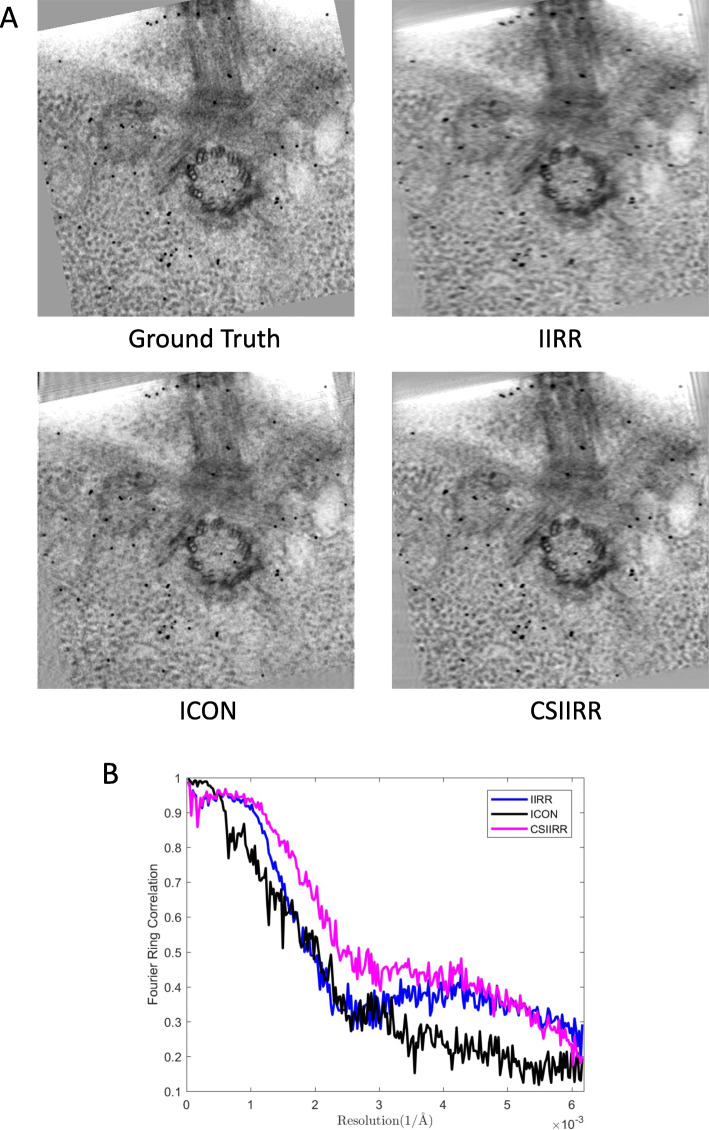
Reprojection result of the Centriole dataset. **a** The excluded projection (groundtruth) and the reprojections at the minimum tilt angle produced by IIRR, ICON and CSIIRR. **b** The FRC curve of IIRR, ICON and CSIIRR, by comparison with the groundtruth

Figure 8 demonstrates the result of reprojection analysis. It can be found that the reprojection produced by IIRR is blurred while the reprojection produced by CSIIRR and ICON are very clear and keep high similarity with the exact excluded groundtruth. The Fourier ring correlation (FRC) curve (Fig. 8b) further indicates that CSIIRR could estimate the missing information more precisely compared with ICON.

#### Experiment on mitochondria

The Mitochondria dataset is a tilt series of mitochondria from mouse hepatic taken with a FEI Tecnai 20, with voltage at 200 kV. cells without fiducial markers. The tilt angles of the projections range from −52.0^∘^ to +59.0^∘^ at 1^∘^ intervals. The tilt series is aligned in advance by the marker-free alignment module in AuTom [16, 21].

**Fig. 9:**
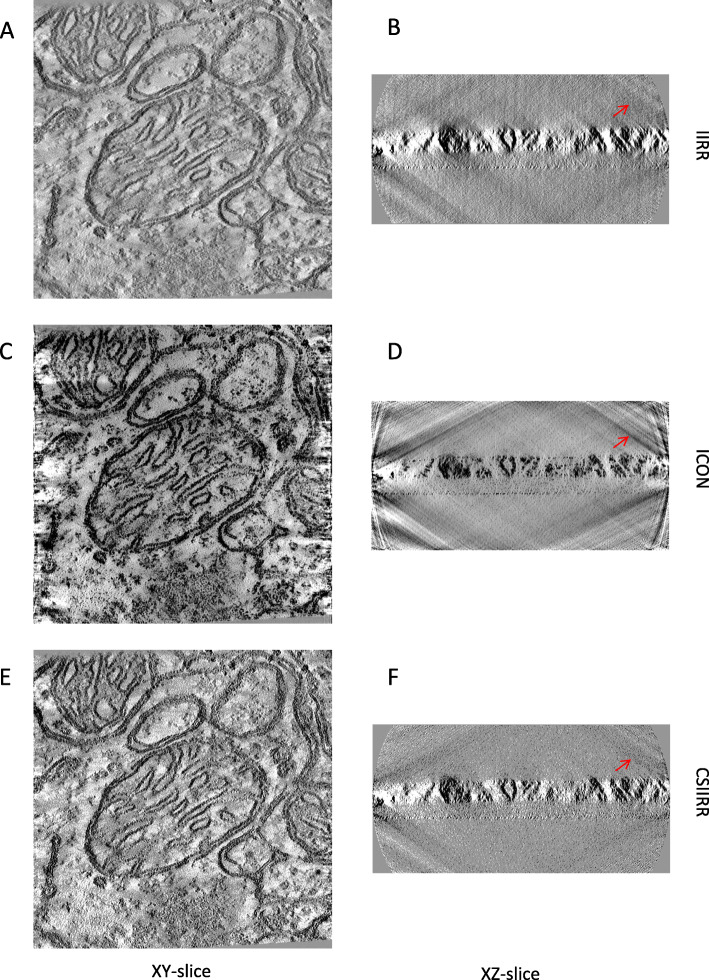
Reconstruction result of the Mitochondria dataset. **a**, **c**, **e** is the XY-slice of reconstruction result from IIRR, ICON and CSIIRR respectively. (B)(D)(F) is the XZ-slice of reconstruction result from IIRR, ICON and CSIIRR respectively

Figure 9 shows the XY-slice and XZ-slice of the reconstructed tomograms. As shown in Fig. 9a, b and e, f, the tomogram reconstructed by CSIIRR has a better contrast comparing with the ones reconstructed by IIRR. According to Fig. 9c, d, stripe noise appears around the boundary of the XY-slice of tomogram from ICON while this kind of stripe noise does not appear around the boundary of the XY-slice of tomogram from CSIIRR.

**Fig. 10:**
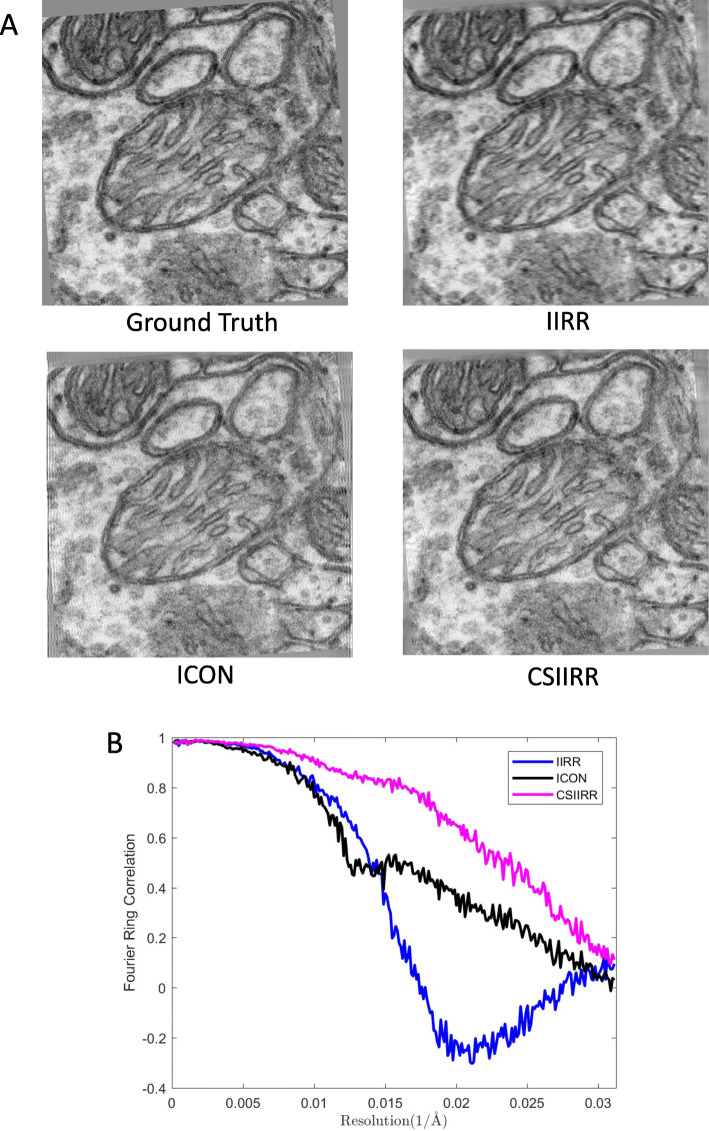
Reprojection result of the Mitochondria dataset. **a** The excluded projection (groundtruth) and the reprojections at the minimum tilt angle produced by IIRR, ICON, and CSIIRR. **b** The FRC curve of IIRR, ICON and CSIIRR, by comparison with the groundtruth

Figure 10 demonstrates the experiment result. As shown in Fig. 10a, the reprojection produced by IIRR has been blurred compared with the groundtruth. The reprojection produced by ICON looks better than IIRR. Nevertheless, strip artifacts still exist around the boundary of the projection produced by ICON. The reprojection produced by CSIIRR has the most visual details and is the most similar to the original excluded projection. The FRC curves shown in Fig. 10b further support our conclusion.

## Conclusion

In this work, we proposed a novel algorithm for the reconstruction of electron tomography. By combining the concept of compressed sensing (CS) and improved iterative reconstruction-reprojection (IIRR) together, this algorithm is able to suppress the effect of the missing wedge and restore the missing information.

Setting a proper value to the parameters in CSIIRR is really important to the performance of this method. First, the factor *λ* should be set carefully to ensure the convergence of the method. The author proposing the IIRR suggested that *λ* should be close to 1, so we set *λ*=0.99 in the experiment. Next, the number of atoms *M* should be set carefully to trade off the convergence rate and the sparsity of the reconstructed volume.

The proposed algorithm was challenged by both the simulated data and real-world data. The results show that the proposed algorithm has an obvious advance in the suppression of missing wedge effects and the restoration of missing information.

## Data Availability

The simulated data were generated from structures at the PDBj (Protein Data Bank Japan) (https://pdbj.org/emnavi/quick.php?id=emdb-3489). The micrograph data are available from the download page of the website of bioinformatics and green network laboratory (http://ear.ict.ac.cn/). Our software implementation is freely available at CSTCloud (https://pan.cstcloud.cn/web/share.html?hash=CQBhG0ViQI8).

## Abbreviations

3D: Three-dimensional
SNR: Signal-to-noise ratio
FBP: Filtered back-projection
TV: Total variation
IRR-TV: Iterative reconstruction-reprojection (IRR) algorithm with total variation (TV) constraint
DIRRLF: Discrete IRR algorithm and the finite impulse response (FIR) lowpass filter
AIRR: Algebraic iterative reconstruction-reprojection
IRRP: Iterative reconstruction-reprojection in projection space
ICON: Iterative compressed-sensing optimized non-uniform fast fourier transform reconstruction
CS: Compressed sensing
NUFFT: Non-uniform fast fourier transform
MP: Matching pursuit
CT: Computed tomography
MMP: Modified MP
PCC: Pearson correlation coefficient
SSIM: Structural similarity
FSC: Fourier shell correlation
FRC: Fourier ring correlation
